# Genomic Analysis Reveals Annual Variation in the Migratory Pathways to East Asia in the Brown Planthopper (*Nilaparvata lugens*)

**DOI:** 10.1111/eva.70171

**Published:** 2025-10-22

**Authors:** Nak Jung Choi, In‐Jeong Kang, Kiwoong Nam

**Affiliations:** ^1^ Crop Environment Research Division National Institute of Crop Science, Rural Development Administration Wanju Republic of Korea; ^2^ DGIMI INRAE, Univ Montpellier Montpellier France

**Keywords:** Brown planthopper, insect population genomics, *Nilaparvata lugens*, pest migratory pathway, wind‐borne pest

## Abstract

The brown planthopper (BPH, *Nilaparvata lugens* ) is a major rice pest in Asia, causing significant yield losses. As BPH cannot overwinter in temperate regions, East Asian populations are wind‐borne migratory insects originating from tropical regions. The identification of precise migratory patterns is essential for forecasting BPH outbreaks and implementing effective pest management strategies. Despite extensive studies using meteorological data, field population observations, and whole‐genome analyses, the BPH migratory pathways to East Asia remain unclear. To address this question, we conducted population genomics analyses using 454 BPH individuals densely collected from China, Korea, and Vietnam between 2017 and 2022. We showed that BPH migration into East Asia exhibits substantial annual variation and involves genomically distinct overwintering origins. Principal component analysis revealed two major groups with whole‐genome differentiation. This separation was confirmed by statistically significant *F*_ST_ estimates, suggesting migration pathways involving at least two overwintering populations. Ancestry coefficient analysis further confirmed the complexity of the ancestry of East Asian BPH. These results demonstrate the complex migratory dynamics of East Asian BPH populations, possibly with the influence of differential selective pressures among overwintering origins. Given the heterogeneity of migratory pathways to East Asia, we argue for temporally and geographically dense field monitoring with the incorporation of genetic information to enhance early warning and BPH management strategies.

## 1 Introduction

The brown planthopper (BPH, *Nilaparvata lugens* ; Delphacidae; Hemiptera) is one of the most serious insect pests affecting rice production in Asia, where 90% of the worldwide rice is produced (Bandumula 2018). Annual losses in rice due to BPH are estimated to be at least 300 million US Dollars in Asia (Min et al. 2014). In particular, occasional outbreaks of BPH have caused severe losses in rice yield (Hu et al. 2014), such as an estimated 1,880,000‐ton loss in 2005 in China (Hu et al. 2011). BPH causes yield losses both directly, by feeding on rice plants, and indirectly, by transmitting virulent viruses such as rice grassy stunt and rice ragged stunt viruses (Cabauatan et al. 2009). BPH management is typically carried out through insecticide spraying, which poses potential ecological and health risks (Ansari et al. 2014). Additionally, insecticide resistance is commonly observed (Datta et al. 2021; Datta and Banik 2021; Wu et al. 2018), adding further complexity to effective pest control strategies.

BPH is distributed across South Asia, East Asia, Southeast Asia, the Pacific islands (Iamba and Dono 2021), and Australia (Claridge et al. 1988). Since BPH can overwinter only in tropical and subtropical regions, such as Southeast Asia and the southernmost parts of China, East Asian BPHs are believed to be wind‐borne migrants from certain southern populations (Iamba and Dono 2021). In line with this observation, laboratory rearing experiments demonstrated that BPH cannot survive at temperatures below 10°C (Noda 1989), which is higher than the average winter temperature in most Chinese territories. BPH migration toward East Asia has been believed to be facilitated by the monsoon (Yang et al. 2022), causing major damage to rice in August. Migrating BPHs have also been directly observed at ocean field stations (Kisimoto 1976) and in traps on airplanes (Deng 1981).

The identification of the exact migratory pathway is critical for forecasting BPH outbreaks and minimizing damage. Therefore, extensive research has been conducted using field population data, meteorological data, and direct observations via radar. These studies have shown that BPHs from Southeast Asia migrate to southern China in spring, then move further north to central and northern China, Korea, and Japan during the monsoon season (reviewed in Iamba and Dono 2021). In the fall, East Asian BPHs migrate southward (Cheng et al. 1979; Hu et al. 2018; Riley et al. 1991), though their final destination remains unclear. The primary overwintering origin of East Asian BPHs is suggested to be North‐Central Vietnam (Hu et al. 2017; Zhang et al. 2025), while other potential sources include Thailand, Laos, and the Philippines (Otuka et al. 2005; Wu et al. 2019; Zhang et al. 2025).

Population genetic analysis based on a single mitochondrial gene has repeatedly shown limitations in inferring population structure (Matsumoto et al. 2013; Mun et al. 1999; Tyagi et al. 2022). For this reason, population genomics studies have been conducted to reveal genetic relationships among geographical populations and gene flow between regions. For example, Hereward et al. (2020) analyzed 63 samples collected between 2009 and 2010 and found that the Chinese BPH populations are genomically undifferentiated from those in the Indochina Peninsula, unlike Philippine BPH populations. Based on these results, they concluded that Chinese BPH populations originated from the Indochina Peninsula. Jeong et al. (2024) analyzed 62 samples from Korea and Southeast Asia between 2020 and 2022 using the Genotyping‐by‐sequencing (GBS) technique, and they reported that Korean BPH populations are genomically differentiated from those in Southeast Asia. They also detected evolutionary signatures of gene flow from Southeast Asia to Korea, an expected pattern even without direct gene flow if long‐term indirect gene flow occurs through overwintering intermediate regions.

Hu et al. (2024) conducted a whole‐genome analysis of 360 samples collected from South Asia, Southeast Asia, East Asia, and Australia between 2009 and 2019. They demonstrated that East Asian BPH populations are genomically differentiated from those in South Asia, Southeast Asia, and Australia, and they suggested that East Asian BPHs are likely to originate from Vietnam, as suggested by field surveys (Hu et al. 2017; Zhang et al. 2025). Intriguingly, Hu et al. reported that genetic differentiation within East Asian BPH populations is higher than in Southeast Asian BPHs, which cannot be readily explained by a simple south–north migration modeling for East Asian BPHs.

In short, the migratory pathways of East Asian BPHs are largely characterized by a simple south‐to‐north movement, although supporting genetic evidence remains sparse, possibly due to insufficient sampling for population genomics analysis in East Asia. To address this question, we performed dense sampling in East Asia, including China, Korea, and Vietnam, from 2017 to 2022, followed by population genomics analysis from 454 samples. This sequencing dataset often includes samples from the same areas across different years, allowing for precise detection of annual variations in migrated BPHs. Here, we identified substantial annual variation in migration pathways toward East Asia, involving multiple genomically differentiated overwintering populations.

## 2 Methods

**FIGURE 1:**
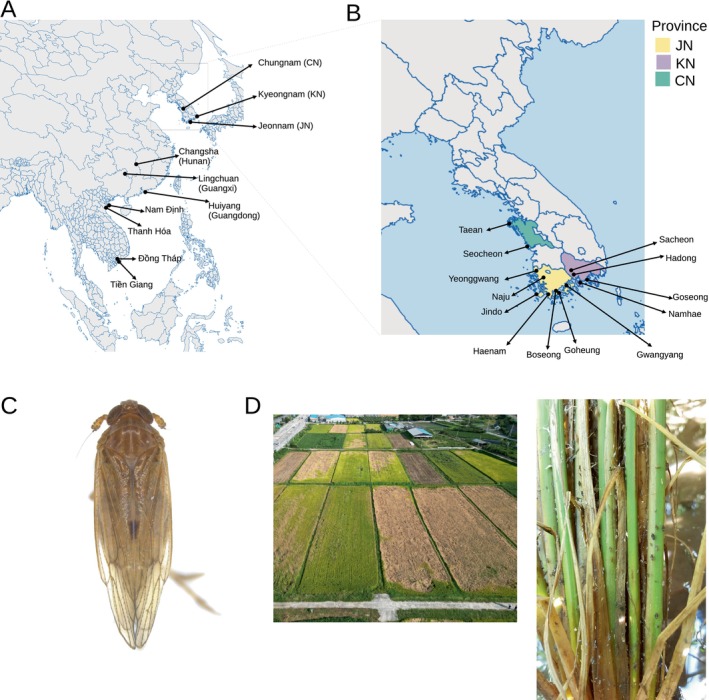
Sampling locations in (A) Asia and (B) a zoomed‐in view of Korea in this study, and (C) the photo of BPH and (D) the damage to rice. The map was generated using MapChart (Voorrips 2002).

Each BPH individual was collected using insect aspirators from three, three, and four provinces in Korea, China, and Vietnam, respectively, between 2017 and 2022 (Table 1, Figure 1A). The Korean samples were obtained from 13 villages across the four provinces (Figure 1B). Multiple samplings were conducted in Gwangyang (16 samples in 2017 and 14 samples in 2018), Goseong (26 in 2020 and 26 in 2022), Namhae (10 in 2017 and 21 in 2022), and Goheung (16 in 2020 and 19 in 2022). This sampling strategy ensures that the samples effectively reveal yearly variations in migration patterns in the Korean Peninsula while controlling for unknown local factors that determine the composition of migrants to specific locations within the peninsula.

**TABLE 1:** The samples used in this study.

Year	Country	Province	Village	Number
2017	China	Guangxi	Lingchuan	29
		Hunan	Changsha	14
	Korea	Jeonnam (JN)	Boseong	21
			Gwangyang	16
			Haenam	15
			Naju	9
			Yeonggwang	25
		Gyeongnam (GN)	Hadong	13
			Namhae	10
2018	China	Guangdong	Huiyang	15
		Guangxi	Lingchuan	8
	Korea	Jeonnam (JN)	Gwangyang	14
2019	China	Guangdong	Huiyang	15
		Hunan	Changsha	15
2020	Korea	Jeonnam (JN)	Goheung	16
		Gyeongnam (GN)	Goseong	26
			Sacheon	18
2022	Korea	Chungnam (CN)	Seocheon	17
			Taean	10
		Jeonnam (JN)	Goheung	19
			Jindo	26
		Gyeongnam (GGN)	Goseong	26
			Namhae	21
	Vietnam	Đồng Tháp	Cao Lãnh	4
		Nam Định	Nam Định	28
		Thanh Hóa	Thanh Hóa	10
		Tiền Giang	My Tho	14

All specimens were immediately placed in 95% ethanol after collection and stored at room temperature. Upon transfer to the laboratory, all samples were stored at −20°C. Genomic DNA was extracted from whole‐body using cetyltrimethylammonium bromide (Allen et al. 2006), followed by dilution of the genomic DNA to 30 ng/μL. Genotyping by sequencing (GBS) was performed based on the protocol of Elshire et al. (2011). About 300 ng of genomic DNA was digested with the restriction enzymes PstI and MspI (New England Biolabs, Ipswich, MA, USA) at 37°C for 3 h. The DNA fragments were then ligated to the adapters with unique barcode sequences to distinguish an individual sample. The ligated DNA samples were purified and amplified by PCR using the following primers: (A) 5′‐AATGATACGGCGACCACCGAGATCTACACTCTTCCCTACACGACGCTCTTCCGATCT and (B) 5′‐CAAGCAGAAGACGGCATACGAGATCGGTCTCGGCATTCCTGCTGAACCGCTCTTCCGATCT. The amplified gDNA samples were pooled and purified using the PCR purification kit (Biofact Inc., Daejeon, South Korea). The resulting GBS libraries were sequenced on an Illumina HiSeqX system.

The pooled GBS reads were demultiplexed and assigned to each sample using the process_radtags function in the STACKS program v 1.38 (Rochette et al. 2019). A total of 97.8% and 95.3% of bases had Phred scores above Q20 and Q30, respectively. Low‐quality bases (Phred quality score < 20) and adaptor sequences in the demultiplexed reads were removed using Trimmomatic v 0.39 (Bolger et al. 2014). The average sequencing coverage per sample of the filtered reads, reported as a standard quality control metric, was calculated using the total sequencing throughput divided by the reference genome size, yielding 0.74 (median = 0.15, range: 0.001 to 15.13), consistent with reduced representation sequencing. The filtered reads were mapped to the *N. lugens* reference genome (Ma et al. 2021) using BWA‐MEM v 0.7.17 (Li and Durbin 2010) with default parameters. The bam files were sorted and indexed using Picard and SAMtools v 1.21 (Li et al. 2009), respectively. Haplotype calling was performed using GATK v4.2.2.0 (McKenna et al. 2010) with the HaplotypeCaller, and variant calling was performed with GenotypeGVCFs. Only single nucleotide polymorphisms were retained from the resulting VCF file, and SNPs were filtered by removing sites with QD < 2.0, FS > 60.0, MQ < 40.0, MQRankSum < −12.5, or ReadPosRankSum < −8.0. Subsequently, we discarded variant positions unless genotypes were determined from at least 50% of the total samples, following a previous study showing that this threshold provides reliable estimates of population genomic statistics for reduced‐representation sequencing data (Marandel et al. 2020). The total number of analyzed single nucleotide polymorphisms was 25,409.

Principal component analysis was conducted using PLINK2 (Purcell et al. 2007), generating ten principal components. Pairwise Weir and Cockerham's *F*_ST_ (Weir and Cockerham 1984) was calculated using VCFtools v0.1.16 (Danecek et al. 2011). Genetic differentiation between groups was statistically tested via a permutation test with 1000 replications, with *p*‐values determined by the proportion of random groupings exhibiting higher *F*_ST_ than the observed grouping. To test whether genetic differentiation between groups occurs only at certain loci or across the whole genome, we calculated pairwise *F*_ST_ in 10 Mb windows across the 1087.8 Mb reference genome. *F*_ST_ values from truncated windows were excluded from the analysis to avoid potential biases due to unequal data sizes between truncated and untruncated windows. Hierarchical *F*_ST_ was calculated using hierfstat v0.5–11 (Goudet 2005).

Targets of selective sweeps were identified using a composite likelihood approach based on the site frequency spectrum with SweeD v4.0.0 (Pavlidis et al. 2013) using 10 grid points per chromosome. Sex chromosomes were excluded due to unequal sample sizes, and the top 1% of composite likelihood values for each group were considered candidate sweep regions.

Gene flow between groups was tested using Treemix v1.13 (Pickrell and Pritchard 2012). For this analysis, a raw fastq file from *N. muiri* was first downloaded from NCBI SRA (Accession number: SRR15665351). Adapter sequences were removed from the reads using adapterremoval (Schubert et al. 2016). Mapping was performed using bowtie v2.3.5.1 (Langmead and Salzberg 2012) with the ‐‐local option, and potential PCR or optical duplicates were removed using picard (2018). Haplotype and variant calling were performed using GATK v4.2.2.0 (McKenna et al. 2010), followed by extraction of biallelic SNPs and filtering according to the criteria of “QD < 2.0 || FS > 60.0 || MQ < 40.0 || MQRankSum < −12.5 || ReadPosRankSum < −8.0”. The resulting VCF file was then merged with the VCF file of BPH using bcftools v1.9 (Danecek et al. 2021). The merged file was converted to a frequency file using vcftools v0.1.16 (Danecek et al. 2011) and PLINK v1.9 (Purcell et al. 2007). A phylogenetic tree incorporating possible gene flow was reconstructed using TreeMix with the number of migration edges set to one.

Ancestry coefficient analysis was conducted using sNMF v1.2, which provides high computational efficiency for large genomic datasets and adaptability to varying sample sizes through adjustment of the regularization parameter (*α*) (Frichot et al. 2014). For this analysis, a PED file was generated from the VCF file of BPH using PLINK v1.9 (Purcell et al. 2007), and subsequently converted to a GENO‐format file with the ped2geno utility of sNMF, which was then used as input for the sNMF program. We tested *α* = 0 and *α* = 100 across *K* values ranging from 1 to 9, and the configuration with the lowest cross‐entropy was selected. Default settings were applied for all other parameters, including the proportion of masked genotypes for cross‐entropy estimation (5%).

## 3 Results

**FIGURE 2:**
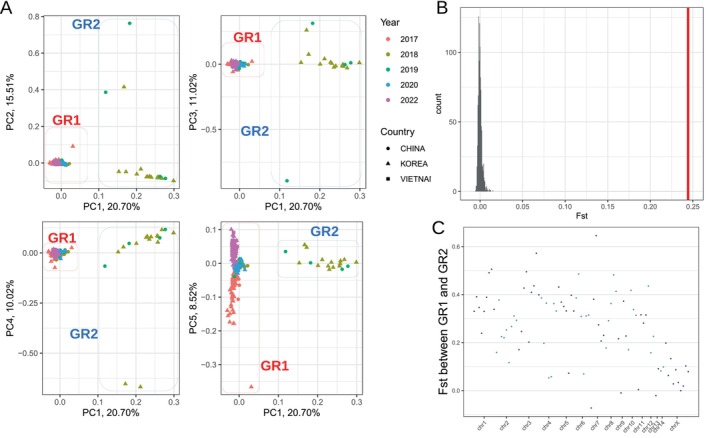
The genomic differentiation among BPH populations. (A) The principal component analysis shows genomic differentiation between GR1 and GR2 along the first principal component. Genomic differentiation among the Group 2 samples according to sampling years starts to be clear from the fifth principal component. (B) *F*_ST_ calculated from GR1 and GR2 is depicted with red vertical bars, and the histogram illustrates *F*_ST_ values calculated with random groupings. (C) *F*_ST_ calculated from untruncated 10 MB windows along the genome indicates genetic differentiation across the whole genome sequence.

Principal component analysis was performed to assess the genetic relatedness among the samples. The first principal component, explaining 20.70% of the total variance, revealed two distinct groups (Figure 2A). The first group (GR1) comprises four out of the 15 samples collected from Guangdong (China) in 2019 and all 14 samples from Gwangyang village (Korea) in 2018. The second group (GR2) consists of the remaining samples. The separation of samples within GR2 based on their respective sampling years started to become clear from the fifth principal component, which explained 8.52% of the variation, whereas separation by geographic location still remained unclear. This pattern was consistent across varying thresholds for the minimum proportion of called genotypes, including the strictest case with no missing data allowed (Figure S1), consistent with a previous study showing that population structure in reduced‐representation sequencing data is robust to this criterion (Chattopadhyay et al. 2014). This result suggests that the grouping into GR1 and GR2 predominantly explains the population structure and that whole‐genome sequences have been differentiated among samples collected in different years to a minor extent.

The genetic differentiation according to geographic locations or sampling years was tested using a hierarchical *F*_ST_ analysis. The hierarchical levels included country, province, and sampling years at the first, second, and third levels, respectively, with the calculated *F*_ST_ values of 0.0183, −0.0033, and 0.0478 at each of these levels. The significance of genetic differentiation at the first and third levels was tested through a permutation test with 1000 replications. The calculated *p*‐values for the first and third levels were 0.016 and 0.001, respectively, indicating statistically supported genetic differentiation according to sampling years as well as sampling countries, consistent with the result of Principal component analysis.

*F*_ST_ calculated between GR1 and GR2 was 0.243 and was significant (permutation test with 1000 replications, *p*‐value < 0.001, Figure 2B). *F*_ST_ along 10 Mb untruncated windows, which contained on average 424.68 SNPs per window (340.09–550.20 of the 95% bootstrap confidence interval) across a total of 88 windows, was greater than zero for 96.6% of the windows (84/87). This result indicates that genetic differentiation between GR1 and GR2 has occurred across the whole genome (Figure 2C).

**FIGURE 3:**
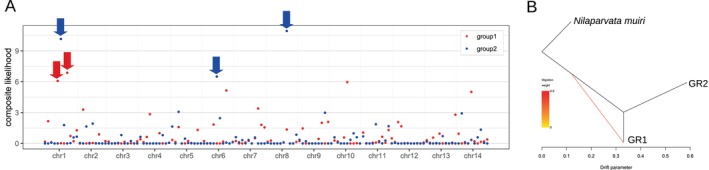
The cause of genetic differentiation between GR1 and GR2. (A) Composite likelihood of being targeted by selective sweeps is shown in red for GR1 and in blue for GR2. The top of 1% composite likelihood outliers are indicated in red (GR1) and blue (GR2) arrows as potential targets of selective sweeps. (B) Phylogenetic tree with gene flow among GR1, GR2, and *Nilaparvata muiri* , as an outgroup. The arrow indicates the inferred direction and magnitude of gene flow.

Selective sweep analysis showed the presence of two loci targeted by the GR1‐specific sweep and three loci targeted by the GR2‐specific sweeps (Figure 3A). TreeMix analysis indicated gene flow from the common ancestor of GR1 and GR2 into the GR1 lineage, whereas no gene flow between the GR1 and GR2 lineages was detected (Figure 3B).

**FIGURE 4:**
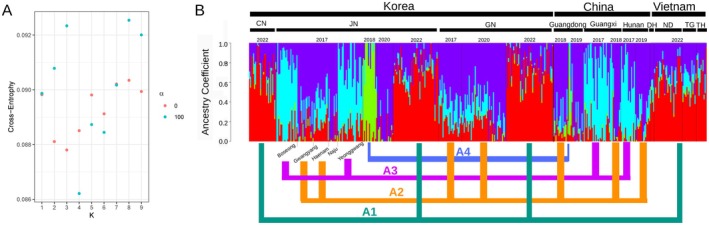
Results of ancestry coefficient analysis. (A) Cross‐entropy values for each *K* with varying *α* (0 or 100). The lowest cross‐entropy was obtained when *K* = 4 and *α* = 100, which we interpreted as indicating four distinct ancestries contributing to the genomic composition of each sample. (B) The samples were classified into four groups (A1–A4) according to their genomic composition of the four ancestries, based on the relative ancestry component of each sample as determined by visual inspection of the sNMF bar plots.

The migratory origins were inferred using ancestry coefficient analysis. The cross‐entropy was lowest when *K* = 4 and *α* = 100, and we chose this result to infer the pattern of ancestry (Figure 4A). In total, we classified the samples into four groups with distinct ancestry patterns (A1–A4, Figure 4) based on the relative ancestry component of each sample, as determined by visual inspection. The Vietnamese samples collected in 2022 shared the same ancestry pattern as the Korean villages in 2022 (A1). The remaining ancestral groups (A2–A4) did not show any pattern according to sampling years, while all samples from GR1 exhibited a shared origin (A4). Intriguingly, despite being only approximately 50 km apart, samples from Gwangyang village in 2017 had a different origin (A2) compared to those from Boseong village in the same year (A3). Assuming that whole‐genome sequences cannot be homogeneously admixed across all samples within just a few generations between migratory arrival and sampling, this result suggests the existence of multiple and complex migratory pathways to East Asia involving the four distinct migrated ancestry groups (A1–A4).

## 4 Discussion

Understanding the trajectory of pest migration is crucial for forecasting the movement of wind‐borne pests (Mazzi and Dorn 2012). Therefore, identifying the BPH migration pattern into East Asia is vital for minimizing rice yield losses and insecticide use through effective early warnings of BPH outbreaks. Genetic data can provide valuable insights by revealing the connectivity of movement among investigated populations. In this study, through population genomics analysis of 454 densely sampled individuals from East Asia and Vietnam, we showed that the pattern of genomic differentiation is best explained by differences among sampled years rather than by sampling locations. This result implies, first, that there are significant annual variations in migratory pathways in East Asia, and second, that multiple distinct overwintering origins with whole genome differentiation contribute to migration to East Asia with complex migratory patterns. Taken together, these results demonstrate the complexity of migration dynamics in East Asia, involving annual variations of migratory pathways and origins.

More specifically, the investigated samples reveal a clear pattern of whole genome differentiation into two groups, GR1 and GR2. GR1 consists of four samples from Guangdong (China) in 2019 and all 14 samples from Gwangyang village (Korea) in 2018, suggesting that these two groups share a common migratory origin. All other samples from GR2 have different origins from GR1, indicating the presence of multiple migratory pathways involving genomically differentiated origins between GR1 and GR2. It remains unclear whether GR1 (or, less likely, GR2 due to the larger sample sizes) is similar to the BPH populations from South Asia or Australia, as these populations have been shown to be genomically distinct from the populations in East Asia (Hereward et al. 2020; Hu et al. 2024).

The genomically differentiated migratory origins imply that different migratory populations may experience distinct selective pressures, as observed in GR1‐ or GR2‐specific selective sweeps (Figure 3A). The observed differentiation across the entire genome sequences between GR1 and GR2 (Figure 2C), without detectable gene flow (Figure 3B), suggests two possibilities. First, the effective migration rate may have been suppressed through the genome hitchhiking effect (Feder et al. 2012). In other words, divergent selection specific to GR1 or GR2 has been sufficiently strong to restrict gene flow across the whole genome sequences, such that the selection coefficient is higher than the migration rate. Second, the overwintering populations of GR1 and GR2 might be allopatrically separated without detectable migration. These two possibilities remain speculative, and the results of the selective sweep analysis need to be interpreted carefully when using reduced‐representation sequencing data due to missing positions. Intriguingly, we also detected a migration edge pointing to GR1 from the common ancestor of GR1 and GR2. The most parsimonious explanation is historical gene flow from an extinct or unsampled population. An alternative, though not mutually exclusive, explanation is the existence of demographic non‐equilibrium, such as ancestral population structure or subdivision. Future studies should focus on characterizing the genomically differentiated populations and inferring the evolutionary mechanisms of group‐specific selection.

It is likely that genetically differentiated overwintering populations have yet to be fully identified, as there is no reason to believe that all Eastern BPHs originate solely from the GR1 or GR2 migratory origins we identified. It is possible that more diverse migratory origins could be uncovered with larger sample sizes. Diverse migratory origins may explain why Hu et al. (2024) observed that East Asian migrants have higher genetic diversity than overwintering BPHs in Southeast Asia. Future studies should focus on Indochina populations, increasing both sample sizes and geographic locations, ideally over a longer time scale.

In this study, we demonstrated that BPH migration in East Asia involves annual variations in patterns and multiple overwintering origins. We assert that this heterogeneity should be considered alongside genomic data for early warning systems of BPH outbreaks. The diverse genomic sequences in the analyzed East Asian BPHs also emphasize the importance of genetic analysis, as different genomic sequences may be associated with different traits, such as fecundity or insecticide resistance. Therefore, we argue that BPH monitoring in East Asia should involve genetic or even genomic characterization. Moreover, sharing information not only among East Asian countries but also with Southeast Asian nations, where BPHs overwinter, is essential, underscoring the importance of coordinated efforts between international phytosanitary agencies.

## Ethics Statement

The authors have nothing to report.

## Conflicts of Interest

The authors declare no conflicts of interest.

## Supporting information


**Figure S1:** Principal component analysis with varying filters according to different thresholds of missing genotypes. The number of retained single nucleotide polymorphisms is also shown above each pane.

## Data Availability

The raw resequencing data used in this study is available at NCBI SRA (Project ID: PRJNA1245615). Computer programming scripts used in this study are available at GitHub (https://github.com/kiwoong‐nam/Nlugens_GBS). The used single nucleotide polymorphism data is available at https://figshare.com/s/60cbd16b57aa0ebf403a. The sampling in China was carried out under the framework of the ‘Cooperative Project on Forecasting of Rice Migratory Pests and Exotic Diseases among China, Vietnam, and Korea.’

## References

[eva70171-bib-0001] Allen, G. C. , M. A. Flores‐Vergara , S. Krasynanski , S. Kumar , and W. F. Thompson . 2006. “A Modified Protocol for Rapid DNA Isolation From Plant Tissues Using Cetyltrimethylammonium Bromide.” Nature Protocols 1, no. 5: 2320–2325. 10.1038/nprot.2006.384. 17406474

[eva70171-bib-0002] Ansari, M. S. , M. A. Moraiet , and S. Ahmad . 2014. “Insecticides: Impact on the Environment and Human Health.” In Environmental Deterioration and Human Health, edited by A. Malik , E. Grohmann , and R. Akhtar , 99–123. Springer Netherlands. 10.1007/978-94-007-7890-0_6.

[eva70171-bib-0003] Bandumula, N. 2018. “Rice Production in Asia: Key to Global Food Security.” Proceedings of the National Academy of Sciences, India Section B: Biological Sciences 88, no. 4: 1323–1328. 10.1007/s40011-017-0867-7.

[eva70171-bib-0004] Bolger, A. M. , M. Lohse , and B. Usadel . 2014. “Trimmomatic: A Flexible Trimmer for Illumina Sequence Data.” Bioinformatics 30, no. 15: 2114–2120. 10.1093/bioinformatics/btu170. 24695404 PMC4103590

[eva70171-bib-0005] Cabauatan, P. Q. , R. C. Cabunagan , and I.‐R. Choi . 2009. “Rice Viruses Transmitted by the Brown Planthopper *Nilaparvata lugens* Stål.” In Planthoppers: New Threats to the Sustainability of Intensive Rice Production Systems in Asia, 357–368. International Rice Research Institute.

[eva70171-bib-0006] Chattopadhyay, B. , K. M. Garg , and U. Ramakrishnan . 2014. “Effect of Diversity and Missing Data on Genetic Assignment With RAD‐Seq Markers.” BMC Research Notes 7: 841. 10.1186/1756-0500-7-841. 25424532 PMC4256836

[eva70171-bib-0007] Cheng, H. , J. Chen , H. Hsi , et al. 1979. “Studies on the Migrations of Brown Planthopper *Nilaparvata lugens* Stal.” https://www.semanticscholar.org/paper/Studies‐on‐the‐migrations‐of‐brown‐planthopper‐Stal‐Cheng‐Chen/ea40e9729d32f4f6cd4a96b51b15e498991d8041.

[eva70171-bib-0008] Claridge, M. F. , J. D. Hollander , and J. C. Morgan . 1988. “Variation in Hostplant Relations and Courtship Signals of Weed‐Associated Populations of the Brown Planthopper, *Nilaparvata lugens* (Stål), from Australia and Asia: A Test of the Recognition Species Concept.” Biological Journal of the Linnean Society 35, no. 1: 79–93. 10.1111/j.1095-8312.1988.tb00460.x.

[eva70171-bib-0009] Danecek, P. , A. Auton , G. Abecasis , et al. 2011. “The Variant Call Format and VCFtools.” Bioinformatics 27, no. 15: 2156–2158. 21653522 10.1093/bioinformatics/btr330 PMC3137218

[eva70171-bib-0010] Danecek, P. , J. K. Bonfield , J. Liddle , et al. 2021. “Twelve Years of SAMtools and BCFtools.” GigaScience 10, no. 2: 1–4. 10.1093/gigascience/giab008. PMC7931819 33590861

[eva70171-bib-0011] Datta, J. , and S. C. Banik . 2021. “Insecticide Resistance in the Brown Planthopper, *Nilaparvata lugens* (Stål): Mechanisms and Status in Asian Countries.” Journal of the Entomological Research Society 23, no. 3: 3. 10.51963/jers.v23i3.2016.

[eva70171-bib-0012] Datta, J. , Q. Wei , Q. Yang , et al. 2021. “Current Resistance Status of the Brown Planthopper *Nilaparvata lugens* (Stål) to Commonly Used Insecticides in China and Bangladesh.” Crop Protection 150: 105789. 10.1016/j.cropro.2021.105789.

[eva70171-bib-0013] Deng, W. X. 1981. “A General Survey on Seasonal Migrations of *Nilaparvata lugens* (Stål) and *Sogatella furcifera* (Horváth)(Homoptera: Delphacidae) by Means of Airplane Collections.” Acta Phytophylacica Sinica 8, no. 2: 73–81.

[eva70171-bib-0014] Elshire, R. J. , J. C. Glaubitz , Q. Sun , et al. 2011. “A Robust, Simple Genotyping‐By‐Sequencing (GBS) Approach for High Diversity Species.” PLoS One 6, no. 5: e19379. 10.1371/journal.pone.0019379. 21573248 PMC3087801

[eva70171-bib-0015] Feder, J. L. , R. Gejji , S. Yeaman , and P. Nosil . 2012. “Establishment of New Mutations Under Divergence and Genome Hitchhiking.” Philosophical Transactions of the Royal Society, B: Biological Sciences 367, no. 1587: 461–474. 10.1098/rstb.2011.0256. PMC3233718 22201175

[eva70171-bib-0016] Frichot, E. , F. Mathieu , T. Trouillon , G. Bouchard , and O. François . 2014. “Fast and Efficient Estimation of Individual Ancestry Coefficients.” Genetics 196, no. 4: 973–983. 10.1534/genetics.113.160572. 24496008 PMC3982712

[eva70171-bib-0017] Goudet, J. 2005. “Hierfstat, a Package for r to Compute and Test Hierarchical *F*‐Statistics.” Molecular Ecology Notes 5, no. 1: 184–186. 10.1111/j.1471-8286.2004.00828.x.

[eva70171-bib-0018] Hereward, J. P. , X. Cai , A. M. A. Matias , G. H. Walter , C. Xu , and Y. Wang . 2020. “Migration Dynamics of an Important Rice Pest: The Brown Planthopper ( *Nilaparvata lugens* ) Across Asia—Insights From Population Genomics.” Evolutionary Applications 13, no. 9: 2449–2459. 10.1111/eva.13047. 33005233 PMC7513714

[eva70171-bib-0019] Hu, C. , J. Guo , X. Fu , Y. Huang , X. Gao , and K. Wu . 2018. “Seasonal Migration Pattern of *Nilaparvata lugens* (Hemiptera: Delphacidae) Over the Bohai Sea in Northern China.” Journal of Economic Entomology 111, no. 5: 2129–2135. 10.1093/jee/toy163. 30010982

[eva70171-bib-0020] Hu, G. , X. N. Cheng , G. J. Qi , et al. 2011. “Rice Planting Systems, Global Warming and Outbreaks of *Nilaparvata lugens* (Stål).” Bulletin of Entomological Research 101, no. 2: 187–199. 10.1017/S0007485310000313. 20961467

[eva70171-bib-0021] Hu, G. , F. Lu , B.‐P. Zhai , et al. 2014. “Outbreaks of the Brown Planthopper *Nilaparvata lugens* (Stål) in the Yangtze River Delta: Immigration or Local Reproduction?” PLoS One 9, no. 2: e88973. 10.1371/journal.pone.0088973. 24558459 PMC3928339

[eva70171-bib-0022] Hu, G. , M.‐H. Lu , H. A. Tuan , et al. 2017. “Population Dynamics of Rice Planthoppers, *Nilaparvata lugens* and *Sogatella furcifera* (Hemiptera, Delphacidae) in Central Vietnam and Its Effects on Their Spring Migration to China.” Bulletin of Entomological Research 107, no. 3: 369–381. 10.1017/S0007485316001024. 27919313

[eva70171-bib-0023] Hu, Q.‐L. , J.‐C. Zhuo , G.‐Q. Fang , et al. 2024. “The Genomic History and Global Migration of a Windborne Pest.” Science Advances 10, no. 17: eadk3852. 10.1126/sciadv.adk3852. 38657063 PMC11042747

[eva70171-bib-0024] Iamba, K. , and D. Dono . 2021. “A Review on Brown Planthopper ( *Nilaparvata lugens* Stål), a Major Pest of Rice in Asia and Pacific.” Asian Journal of Research in Crop Science 7–19: 7–19. 10.9734/ajrcs/2021/v6i430122.

[eva70171-bib-0025] Jeong, S. , H. Gill , T. Yu , et al. 2024. “A Genomic Investigation on the Origins of the Korean Brown Planthopper, *Nilaparvata lugens* (Stål) (Hemiptera: Delphasidae).” Entomological Research 54, no. 5: e12722. 10.1111/1748-5967.12722.

[eva70171-bib-0026] Kisimoto, R. 1976. “Synoptic Weather Conditions Inducing Long‐Distance Immigration of Planthoppers, *Sogatella furcifera* Horvath and *Nilaparvata lugens* Stal.” Ecological Entomology 1, no. 2: 95–109. 10.1111/j.1365-2311.1976.tb01210.x.

[eva70171-bib-0027] Langmead, B. , and S. L. Salzberg . 2012. “Fast Gapped‐Read Alignment With Bowtie 2.” Nature Methods 9, no. 4: 357–359. 10.1038/nmeth.1923. 22388286 PMC3322381

[eva70171-bib-0028] Li, H. , and R. Durbin . 2010. “Fast and Accurate Long‐Read Alignment With Burrows–Wheeler Transform.” Bioinformatics 26, no. 5: 589–595. 20080505 10.1093/bioinformatics/btp698 PMC2828108

[eva70171-bib-0029] Li, H. , B. Handsaker , A. Wysoker , et al. 2009. “The Sequence Alignment/Map Format and SAMtools.” Bioinformatics 25, no. 16: 2078–2079. 19505943 10.1093/bioinformatics/btp352 PMC2723002

[eva70171-bib-0030] Ma, W. , L. Xu , H. Hua , et al. 2021. “Chromosomal‐Level Genomes of Three Rice Planthoppers Provide New Insights Into Sex Chromosome Evolution.” Molecular Ecology Resources 21, no. 1: 226–237. 10.1111/1755-0998.13242. 32780934

[eva70171-bib-0031] Marandel, F. , G. Charrier , J. Lamy , S. Le Cam , P. Lorance , and V. M. Trenkel . 2020. “Estimating Effective Population Size Using RADseq: Effects of SNP Selection and Sample Size.” Ecology and Evolution 10, no. 4: 1929–1937. 10.1002/ece3.6016. 32128126 PMC7042749

[eva70171-bib-0032] Matsumoto, Y. , M. Matsumura , S. Sanada‐Morimura , Y. Hirai , Y. Sato , and H. Noda . 2013. “Mitochondrial Cox Sequences of *Nilaparvata lugens* and *Sogatella furcifera* (Hemiptera, Delphacidae): Low Specificity Among Asian Planthopper Populations.” Bulletin of Entomological Research 103, no. 4: 382–392. 10.1017/S000748531200082X. 23537548

[eva70171-bib-0033] Mazzi, D. , and S. Dorn . 2012. “Movement of Insect Pests in Agricultural Landscapes.” Annals of Applied Biology 160, no. 2: 97–113. 10.1111/j.1744-7348.2012.00533.x.

[eva70171-bib-0034] McKenna, A. , M. Hanna , E. Banks , et al. 2010. “The Genome Analysis Toolkit: A MapReduce Framework for Analyzing Next‐Generation DNA Sequencing Data.” Genome Research 20, no. 9: 1297–1303. 10.1101/gr.107524.110. 20644199 PMC2928508

[eva70171-bib-0035] Min, S. , S. W. Lee , B.‐R. Choi , S. H. Lee , and D. H. Kwon . 2014. “Insecticide Resistance Monitoring and Correlation Analysis to Select Appropriate Insecticides Against *Nilaparvata lugens* (Stål), a Migratory Pest in Korea.” Journal of Asia‐Pacific Entomology 17, no. 4: 711–716. 10.1016/j.aspen.2014.07.005.

[eva70171-bib-0036] Mun, J. H. , Y. H. Song , K. L. Heong , and G. K. Roderick . 1999. “Genetic Variation Among Asian Populations of Rice Planthoppers, *Nilaparvata lugens* and *Sogatella furcifera* (Hemiptera: Delphacidae): Mitochondrial DNA Sequences.” Bulletin of Entomological Research 89, no. 3: 245–253. 10.1017/S000748539900036X.

[eva70171-bib-0037] Noda, H. 1989. “Developmental Zero and Total Effective Temperature of Three Rice Planthoppers (Homoptera: Delphacidae).” Japanese Journal of Applied Entomology and Zoology 33: 263–266. 10.5555/19901146049.

[eva70171-bib-0038] Otuka, A. , T. Watanabe , Y. Suzuki , M. Matsumura , A. Furuno , and M. Chino . 2005. “A Migration Analysis of the Rice Planthopper *Nilaparvata lugens* From The Philippines to East Asia With Three‐Dimensional Computer Simulations.” Population Ecology 47, no. 2: 143–150. 10.1007/s10144-005-0216-1.

[eva70171-bib-0039] Pavlidis, P. , D. Živković , A. Stamatakis , and N. Alachiotis . 2013. “SweeD: Likelihood‐Based Detection of Selective Sweeps in Thousands of Genomes.” Molecular Biology and Evolution 30, no. 9: 2224–2234. 10.1093/molbev/mst112. 23777627 PMC3748355

[eva70171-bib-0040] Picard . 2018. “picard: A Set of Command Line Tools (in Java) for Manipulating High‐Throughput Sequencing (HTS) Data and Formats Such as SAM/BAM/CRAM and VCF [Java].” Broad Institute. https://github.com/broadinstitute/picard. (Original work published 2014).

[eva70171-bib-0041] Pickrell, J. K. , and J. K. Pritchard . 2012. “Inference of Population Splits and Mixtures From Genome‐Wide Allele Frequency Data.” PLoS Genetics 8, no. 11: e1002967. 10.1371/journal.pgen.1002967. 23166502 PMC3499260

[eva70171-bib-0042] Purcell, S. , B. Neale , K. Todd‐Brown , et al. 2007. “PLINK: A Tool Set for Whole‐Genome Association and Population‐Based Linkage Analyses.” American Journal of Human Genetics 81, no. 3: 559–575. 10.1086/519795. 17701901 PMC1950838

[eva70171-bib-0043] Riley, J. R. , C. Xia‐Nian , Z. Xiao‐Xi , et al. 1991. “The Long‐Distance Migration of *Nilaparvata lugens* (Stål) (Delphacidae) in China: Radar Observations of Mass Return Flight in the Autumn.” Ecological Entomology 16, no. 4: 471–489. 10.1111/j.1365-2311.1991.tb00240.x.

[eva70171-bib-0044] Rochette, N. C. , A. G. Rivera‐Colón , and J. M. Catchen . 2019. “Stacks 2: Analytical Methods for Paired‐End Sequencing Improve RADseq‐Based Population Genomics.” Molecular Ecology 28, no. 21: 4737–4754. 10.1111/mec.15253. 31550391

[eva70171-bib-0045] Schubert, M. , S. Lindgreen , and L. Orlando . 2016. “AdapterRemoval v2: Rapid Adapter Trimming, Identification, and Read Merging.” BMC Research Notes 9: 88. 10.1186/s13104-016-1900-2. 26868221 PMC4751634

[eva70171-bib-0046] Tyagi, S. , S. Narayana , R. N. Singh , et al. 2022. “Migratory Behaviour of Brown Planthopper, *Nilaparvata lugens* (Stål) (Hemiptera: Delphacidae), in India as Inferred From Genetic Diversity and Reverse Trajectory Analysis.” 3 Biotech 12, no. 10: 266. 10.1007/s13205-022-03337-6. PMC9458824 36091088

[eva70171-bib-0047] Voorrips, R. E. 2002. “MapChart: Software for the Graphical Presentation of Linkage Maps and QTLs.” Journal of Heredity 93, no. 1: 77–78. 10.1093/jhered/93.1.77. 12011185

[eva70171-bib-0048] Weir, B. S. , and C. C. Cockerham . 1984. “Estimating F‐Statistics for the Analysis of Population Structure.” Evolution 38: 1358–1370. 28563791 10.1111/j.1558-5646.1984.tb05657.x

[eva70171-bib-0049] Wu, Q. , G. Hu , H. A. Tuan , et al. 2019. “Migration Patterns and Winter Population Dynamics of Rice Planthoppers in Indochina: New Perspectives From Field Surveys and Atmospheric Trajectories.” Agricultural and Forest Meteorology 265: 99–109. 10.1016/j.agrformet.2018.11.001.

[eva70171-bib-0050] Wu, S.‐F. , B. Zeng , C. Zheng , et al. 2018. “The Evolution of Insecticide Resistance in the Brown Planthopper ( *Nilaparvata lugens* Stål) of China in the Period 2012–2016.” Scientific Reports 8, no. 1: 4586. 10.1038/s41598-018-22906-5. 29545538 PMC5854692

[eva70171-bib-0051] Yang, S.‐J. , Y.‐X. Bao , X.‐F. Zheng , and J. Zeng . 2022. “Effect of the Asian Monsoon on the Northward Migration of the Brown Planthopper to Northern South China.” Ecosphere 13, no. 10: e4217. 10.1002/ecs2.4217.

[eva70171-bib-0052] Zhang, X.‐Y. , H. Lv , Y.‐Y. Zhang , et al. 2025. “Long‐Term Seasonal Forecasting Model for the Trans‐Regional Migration of Brown Planthopper in Eastern China.” Insect Science 3: 1–13. 10.1111/1744-7917.70013. 40033415

