# Supplementary material for: Genomic Analysis Reveals Annual Variation in the Migratory Pathways to East Asia in the Brown Planthopper (Nilaparvata lugens)
Source: Evol Appl. 2025 Oct 22;18(10):e70171. doi: 10.1111/eva.70171 (PMC12542306; doi:10.1111/eva.70171)
Supplement: Supplementary file 1 — Figure S1: Principal component analysis with varying filters according to different thresholds of missing genotypes. The number of retained single nucleotide polymorphisms is also shown above each pane. [file EVA-18-e70171-s001.docx]

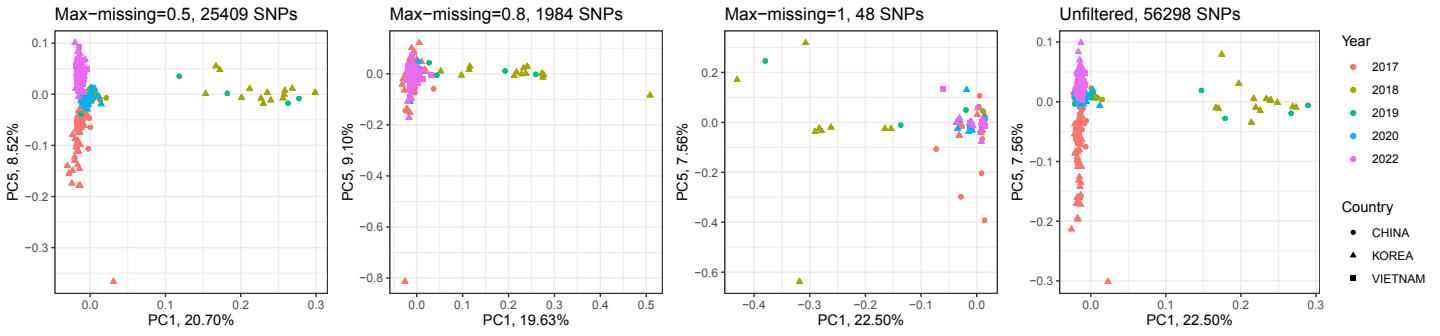
Figure S1. Principal component analysis with varying filters according to different thresholds of missing genotypes. The number of retained single nucleotide variants is also shown above each pane.
